# Intracranial Bleeding After Reperfusion Therapy in Acute Ischemic Stroke

**DOI:** 10.3389/fneur.2020.629920

**Published:** 2021-02-09

**Authors:** Guillaume Charbonnier, Louise Bonnet, Alessandra Biondi, Thierry Moulin

**Affiliations:** ^1^Neurology Department, Besançon University Hospital, Besançon, France; ^2^Interventional Neuroradiology Department, Besançon University Hospital, Besançon, France; ^3^EA 481 Neurosciences laboratory, Franche-Comté University, Besançon, France; ^4^CIC-1431 Inserm, Besançon, France

**Keywords:** stroke, intracranial bleeding, brain hemorrhage, hemorrhagic transformation, reperfusion, intravenous thrombolysis, mechanical thrombectomy

## Abstract

Intracranial hemorrhage is one of the most feared complications following brain infarct. Ischemic tissues have a natural tendency to bleed. Moreover, the first recanalization trials using intravenous thrombolysis have shown an increase in mild to severe intracranial hemorrhage. Symptomatic intracerebral hemorrhage is strongly associated with poor outcomes and is an important factor in recanalization decisions. Stroke physicians have to weigh the potential benefit of recanalization therapies, first, with different risks of intracranial hemorrhage described in randomized controlled trials, and second with numerous risk markers that have been found to be associated with intracranial hemorrhage in retrospective series. These decisions have become quite complex with different intravenous thrombolytics and mechanical thrombectomy. This review aims to outline some elements of the pathophysiological mechanisms and classifications, describe most of the risk factors identified for each reperfusion therapy, and finally suggest future research directions that could help physicians dealing with these complications.

## Introduction

Recent advances in reperfusion therapies for acute ischemic stroke have required stroke physicians to deepen their understanding of cerebral hemorrhagic complications. Although the overall risks have been well-documented in various randomized controlled trials (RCT) of reperfusion therapies, the mechanisms underlying cerebral bleeding for an individual patient are still poorly understood. Intracranial bleeding following acute ischemic stroke has a major impact on patient outcomes, and controlling the risk of bleeding plays an important role in recanalization decisions. Intracranial bleeding can take a wide range of different forms, from extraparenchymal (subdural hematoma and subarachnoid hemorrhage) to intraparenchymal. The latter can be small suffusions to large hematomas with mass effect within infarction, all of which are called hemorrhagic transformation (HT). Large parenchymal hematomas are the most feared due to a high mortality rate and are present in ~6% of patients after intravenous thrombolysis (IVT). In addition, infarct evolution and/or HT can cause dramatic neurologic deterioration. The frequency of HT is dependent on very different factors, including epidemiological factors (age, pre-stroke treatment, and conditions, etc.), characteristics of the infarct (size of ischemic core, timing of follow-up), reperfusion techniques at the acute phase (intravenous thrombolysis, mechanical thrombectomy, etc.), radiological diagnosis (computed tomography, magnetic resonance imaging), and antithrombotics following the acute phase. This review aims to outline some elements of the pathophysiological mechanisms and classifications of HT, describe most of the risk factors identified for each reperfusion therapy, and finally suggest future research directions that could help physicians dealing with these complications.

## Pathophysiological Mechanisms of Intracranial Bleeding Following Brain Infarct

Cerebral infarcts occur when a diminution of cerebral blood flow reaches a minimum threshold at which it cannot ensure a sufficient amount of oxygen and glucose. Like in many other organs, brain ischemic parenchyma have a tendency to bleed, and brain hemorrhage can lead to severe neurological deterioration. Mechanisms involved in hemorrhagic transformation can be considered from various points of view, including histological changes, vascular occlusion, collateral circulation, blood-brain barrier disruption, and infarct size.

Acute cerebral ischemia leads to the death of capillary cells, which causes vascular permeability and extravasation of blood in the brain parenchyma. The two main factors described in this process are oxidative stress and reperfusion injury. These factors lead to various mechanisms such as inflammation, leukocyte infiltration, vascular activation, and extracellular proteolysis (1, 2). The consequences are destruction of the basal lamina and endothelial tight junctions. Among the molecular processes involved, matrix metalloproteinase 9 (MMP-9) has been shown to play an important role in the destruction of basal lamina type IV collagen (1). Destruction of the basal lamina leads to leakage of macromolecules into the brain interstitial fluids. The resulting ionic gradient induces interstitial edema, known as vasogenic edema (as opposed to cytotoxic edema, which is associated with cell death). Vasogenic edema can lead to lesions on adjacent tissues. In the case of large infarcts, this mechanism can worsen and cause a malignant infarct, with catastrophic outcomes and high risk of hemorrhagic transformation. Hemorrhage seems to be very prevalent in ischemic lesions. In fact, early CT studies found that 65% of “pale infarcts,” defined as negative CT for blood after ischemic stroke, showed petechial hemorrhage on microscopic sections (3). Reperfusion is involved in various pathways leading to cerebral injuries (4). First, it worsens ischemic injuries such as oxidative stress, suppression of protein synthesis, platelet activation, activation of the complement system, leukocyte infiltration, basal lamina disruption, and cerebral cell death. Moreover, reperfusion could induce specific mechanisms such as secondary hyperperfusion and hypoperfusion. The quantitative roles of thrombotic and inflammatory processes vs. reperfusion injuries are debated (5). Microvascular thromboinflammation could be hard to differentiate from reperfusion injuries.

Although ischemic lesions seem to be enough to cause hemorrhagic injuries, it has been suggested that late fragmentation of a thrombus, especially a larger one, could be the cause of late hemorrhagic complications. Fragmentation of a large thrombus could lead to distal migration and could damage the vascular bed (3).

It has been questioned if an occluded artery that causes a reduction of blood flow could decrease the risk of HT by reducing reperfusion injuries. The possibility of hemorrhagic complication with persistence of a proximal occlusion has led to discussion about the role of collateral circulation from the leptomeningeal network. A first cadaveric study demonstrated the presence of hemorrhagic infarcts with persistent proximal occlusion and possible involvement of the leptomeningeal network in reperfusion injuries (6). Another study, which used computed tomography to show hemorrhagic transformation and repeated angiography to demonstrate persistent occlusion and collateral development, highlighted a case of cortical HT in relation to collateral development (7).

On the other hand, reperfusion of the cerebral infarct could reduce ischemia and therefore reduce the risk of hemorrhage. Thus, higher prevalence of bleeding in the lenticulostriate artery territories could be linked to the absence of collateral anastomosis. It takes the form of a hemorrhage inside the infarcted tissue corresponding to intra-infarct hematoma or parenchymal hematoma. Profound ischemia leads to endothelial necrosis and hematomas (3). Additionally, increased risk of hemorrhage is well-documented in large infarcts, which supports the hypothesis of necrosis having a preponderant role in hemorrhage risk.

Another possible mechanism could be an abnormal response of the arterial wall to brain ischemia. Vasospasm is a complex phenomenon that is observed after the smooth muscular fibers of the vascular wall have been damaged. Hemorrhagic infarcts have been documented following vasospasm, so this could be another mechanism for hemorrhage following reperfusion (8). Furthermore, intimal lesions have been observed on occluded arteries. It has been debated whether these lesions were direct damage from the clot or consequences of secondary vasospasm (9).

Finally, hemorrhage in infarcted tissues seems to appear in a relatively short time window after stroke. Indeed, this complication occurs mostly within 24 h of thrombolysis (10).

## Reperfusion Therapies and Hemorrhagic Transformation: Learning From Intravenous Thrombolysis, Intra-Arterial Thrombolysis, Mechanical Thrombectomy, and Sonothrombolysis

Before reperfusion therapies, hemorrhagic transformation of acute ischemic stroke was not recognized and was badly classified. In fact, HT was usually described in the context of available treatments, mostly antithrombotics including anticoagulants. Use of reperfusion techniques in the acute phase of stroke has led to new needs for classification in RCTs.

### Brief Historical Insight Into Hemorrhagic Transformation Related to Reperfusion Therapies

Before describing intracranial bleeding, it is necessary to review each reperfusion therapy and its latest developments. The hemorrhagic risk of these therapies is detailed in these therapies is detailed further. In the mid-1990s, the MAST-E and MAST-I trials failed to demonstrate the efficacy of intravenous streptokinase (11, 12) in the 6-h therapeutic window and showed an increased risk of HT and death in the intervention group. The ECASS I and II trials using tPA in the 6-h therapeutic window were also negative, with an increased rate of HT (13). The NINDS (14) trial showed the benefit of intravenous infusion of tPA in the 3-h therapeutic window, but with an increased risk of intracerebral hemorrhage in the intervention group. The large retrospective study SITS-MOST confirmed randomized data with real-life experience (15). Superiority of tPA in terms of functional independence at 3 months was then further demonstrated in the ECASS III (16) trial for the 4.5-h therapeutic window. Although negative on its primary outcome, IST-3 showed a significant shift on the Oxford Handicap Score using tPA in the 6-h therapeutic window, including in patients over 80 years old (17). The intervention was also associated with an increased risk of HT. Interestingly, mortality increased for the 7 days following IVT, but the rate was reversed between 7 days and 6 months so that similar rates were observed at 6 months. The therapy consisted of a 1-h intravenous infusion of alteplase, a fibrinolytic therapy, in order to achieve clot lysis and brain reperfusion. In 1998, a first phase II trial used intra-arterial thrombolysis as a new reperfusion technique (18). The investigators tried to achieve thrombus lysis by infusing recombinant pro-urokinase through a microcatheter placed in the thrombus or the M1 segment of the middle cerebral artery. Increased intracerebral hemorrhage was observed in the intervention group. In 2004, the first RCT using sonothrombolysis for cerebral recanalisation using transcranial 2-MHz Doppler Ultrasound showed a similar hemorrhagic risk. In 2015, five RCTs demonstrated the superiority of MT in anterior circulation acute ischemic stroke caused by large vessel occlusion (19), followed by two others (20, 21). The intervention consisted of an endovascular procedure, with catheterization of the cervical carotid and then the middle cerebral artery. The clot was removed mostly with the use of a stent retriever device. This consists of deploying a temporary stent delivered by a microcatheter to the site of occlusion. The clot is captured by the stent strands and then the stent is removed with the clot, achieving good recanalization in 80% of cases. The intervention group did not experience a higher incidence of intracranial bleeding. There are specific causes of bleeding after MT, as MT devices could led to endothelial lesions. Vessel wall components are found on histological analyses of thrombi (22), and vessel wall enhancement is found on the follow-up MRI after MT (23). The procedure may require microcatheterization with microwire, which could cause direct endothelial lesions and, rarely, perforation (24).

### Diagnosis and Classifications

#### Radiological Screening of Hemorrhage After Cerebral Infarct

Surprisingly, there are no recommendations for the type of brain imaging or delay after reperfusion therapy. The ECASS III protocol performed a CT or MRI 22–36 h after IVT (16). In addition, there are no recommendations for a clear screening protocol for HT in the subacute phase for patients receiving different antithrombotic treatments. Also, no radiological patterns have been identified that could guide patient management. For now, only neurological deterioration is a clear indication for emergency HT screening.

#### Neuro-Imaging Classifications

**Figure 1:**
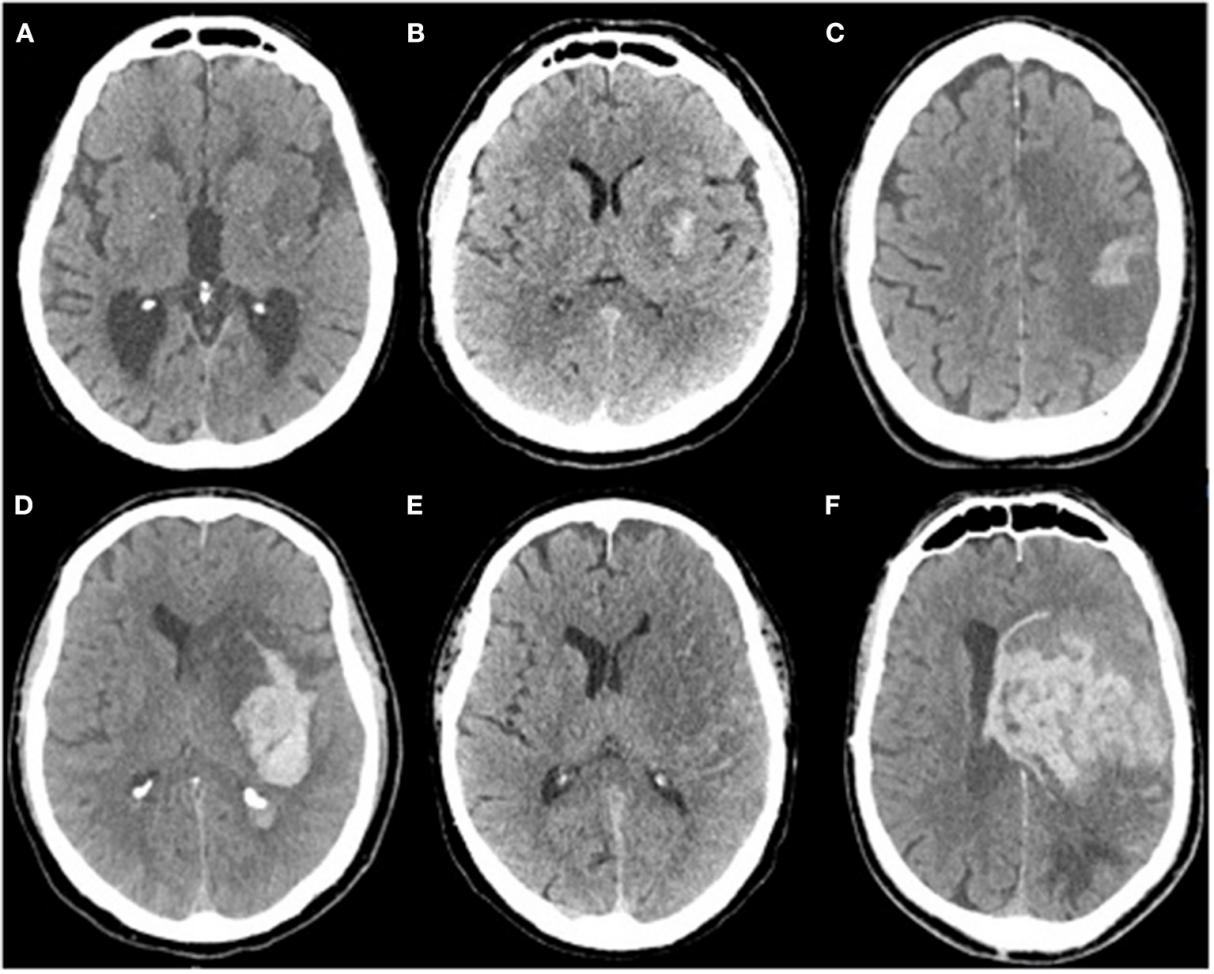
**(A)** Isolated petechia of the posterior part of the lenticular nucleus: Moulin Hi d1, ECASS HI1, Heidelberg HI1, **(B)** Confluent petechial of the lenticular nucleus: Moulin HI d2, ECASS HI2, Heidelberg HI 2, **(C)** Cortical parenchymal hemorrhage: Moulin HI c1, ECASS HI1, Heidelberg HI1, **(D)** Deep parenchymal hemorrhage with mass effect and intraventricular hemorrhage: Moulin IIH d2, ECASS PH2, Heidelberg PH2 + class 3b, **(E)** isolated subarachnoid hemorrhage: Heidelberg class 3c, **(F)** Massive parenchymal hematoma Moulin IIH d2, ECASS, and Heidelberg PH2.

**Figure 2:**
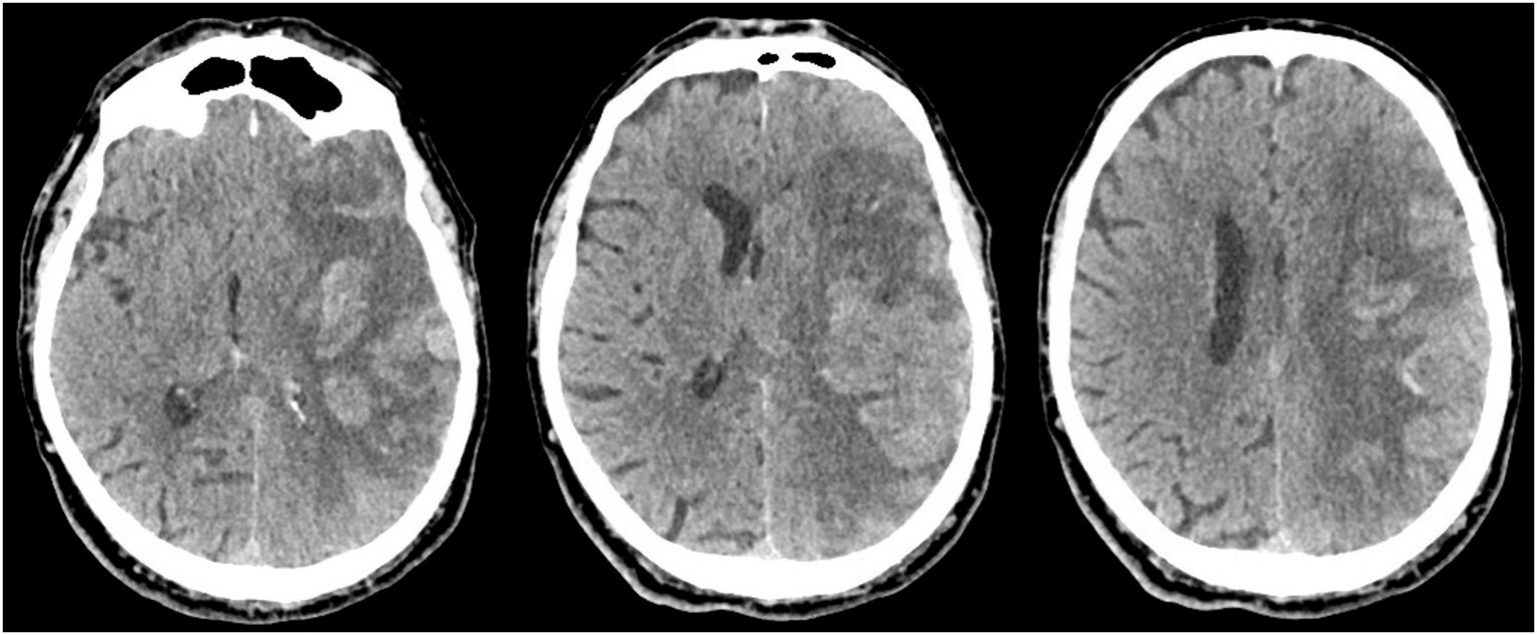
Massive cortical hemorrhage with mass effect mostly caused by the infarcted tissue, which could be classified as Moulin HI c2, ECASS HI2, or Heidelberg HI2.

Intracranial bleeding after acute ischemic stroke can follow various radiological patterns. A few classifications have been proposed, first every ischemic stroke (Table 1) (3), and then simplified for thrombolysis RCTs (13). The ECASS grading system (Table 2) differentiates Parenchymal Hematoma (PH) from Hemorrhagic Infarction (HI). PH1 was differentiated from PH2 by a bleeding volume of <30% of the underlying ischemia (25). A few examples of radiological patterns classified by different methods can be seen in Figure 1. Even if the different radiological classifications are quite similar, particularly regarding the difference between PH and HI, some situations can be more challenging to classify (Figure 2).

**Table 1:** Adapted from Moulin et al. (3).

**Hemorrhagic Infarct (HI)**		
Cortical	HI c1	Petechial aspect located in the cortex
	HI c2	Confluent aspect involving 50–75% of the vascular territory
Deep	HI d1	Punctiform hemorrhage in the deep vascular territory
	HI d2	Confluent hemorrhage involving 50–75% of the vascular territory
**Intra-Infarct Hematoma (IIH)**		
Cortical	IIH c1	Homogeneous aspect involving 75–100% of the vascular territory without mass effect
	IIH c2	Hemorrhage in the whole vascular territory, liquid level, or mass effect
Deep	IIH d1	Homogeneous aspect involving 75–100% of the deep vascular territory with moderate mass effect
	IIH d2	Massive deep hemorrhage with severe mass effect

**Table 2:** ECASS classification, adapted from Larrue et al. (25).

PH1	Blood clots in ≤ 30% of the infarcted area with some slight space-occupying effect
PH2	Blood clots in >30% of the infarcted area with a substantial space-occupying effect
HI1	Small petechiae along the margins of the infarct
HI2	Confluent petechiae within the infarcted area but no space-occupying effect

The latter classification, which is used mostly for retrospective MT studies, was re-defined by a team of experts including investigators from the first MT RCTs, who proposed the Heidelberg Bleeding Classification (HBC) (26) (Table 3). A definite symptomatic intracerebral hemorrhage (sICH) is defined as a PH2 hematoma with a significant clinical deterioration (four points on NIHSS or two points in one category) if the bleeding is the main cause of deterioration. In this case, there is no other dominant cause to explain the clinical deterioration (e.g., a partially hemorrhagic malignant infarct). Moreover, the HBC introduced new categories for previously non-classified intracerebral hemorrhages (intraventricular, subarachnoid, and subdural hemorrhages) and provided a formal approach to classifying those hemorrhages. These additions mean that substantially more hemorrhages can be diagnosed using the HBC compared to the ECASS classification (27).

**Table 3:** Heidelberg bleeding classification, adapted from von Kummer et al. (26).

**Class**	**Type**	**Description**
1	Hemorrhagic transformation of infarcted brain tissue	
1a	HI1	Scattered small petechiae, no mass effect
1b	HI2	Confluent petechiae, no mass effect
1c	PH1	Hematoma within infarcted tissue, occupying <30%, no substantive mass effect
2	Intracerebral hemorrhage within and beyond infarcted brain tissue	
	PH2	Hematoma occupying 30% or more of the infarcted brain tissue, with obvious mass effect
3	Intracerebral hemorrhage outside the infarcted brain tissue or intracranial-extracerebral hemorrhage	
3a	Parenchymal hematoma remote from infarcted brain tissue	
3b	Intraventricular hemorrhage	
3c	Subarachnoid hemorrhage	
3d	Subdural hemorrhage	

### Prognosis and Management of Hemorrhagic Transformation

sICH has been shown to be a catastrophic complication of acute ischemic stroke, with poorer clinical outcomes at 3 months (28–30). Only PH2 hemorrhages were initially associated with poor outcomes in ECASS. Recent retrospective studies also show an association with every ICH other than subarachnoid hemorrhage (SAH) in a MT cohort (31, 32). A retrospective study found an association between poor outcome and type 2 SAH, but not type 1 SAH, after endovascular thrombectomy (33). In that study, type 2 SAH was defined as SAH with isolated intra-parenchymal hematoma or other intracerebral hemorrhage.

Management of hemorrhagic transformation was discussed in the American Heart Association 2017 guidelines (34), which are summarized in Table 4. For alteplase-induced HT, cryoprecipitates, fresh frozen plasma, and tranexamic acid seem to have potential benefits. Blood pressure (BP) objectives should be guided by the recanalization status. In a fully recanalized patient, reduction of BP should be aggressive. Decompressive hemicraniectomy may be considered in select patients with sICH for whom surgery may improve outcome despite the ischemic injury.

**Table 4:** Suggestions for reversal agents that may be considered on the basis of the mechanisms of action of the agent and alteplase in patients with sICH occurring within 36 h after alteplase infusion, adapted from Yaghi et al. (34).

**Reversal agent**	**Suggested dose**	**Potential for benefit**	**Adverse effects**
Cryoprecipitate	Consider sending a fibrinogen level immediately and empirically transfusing with 10 U cryoprecipitate, and anticipate giving more cryoprecipitate as needed to achieve a normal fibrinogen level of ≥150 mg/dL (10 U cryoprecipitate increases fibrinogen by nearly 50 mg/dL)	Potential for benefit in all sICH	Transfusion reaction and transfusion-related lung injury
Platelets	2 donors (8–10 U)	Potential for benefit is unclear except in patients with thrombocytopenia (platelets <100,000/μL), who may possibly benefit	Transfusion reaction, transfusion-related lung injury, volume overload
FFP	12 mL/kg	Potential for benefit is unclear except in patients on warfarin, in whom FFP may be considered	Transfusion reaction, transfusion-related lung injury, volume overload
PCC	25–50 U/kg (based on INR level)	Potential for benefit is unclear except in patients on warfarin, in whom PCC may be considered and is the preferred adjunctive treatment	Thrombotic complications
Vitamin K	10 mg intravenously	Potential for benefit is unclear except in patients on warfarin, in whom vitamin K may be used as an adjunctive treatment	Anaphylaxis
rFVIIa	20–160 μg/kg	Potential for benefit is unclear	Thrombotic complications
Antifibrinolytic agents	Aminocaproic acid: 4 g IV during first hour followed by 1 g/h for 8 h Tranexamic acid: 10 mg/kg 3–4 times/d (adjustment based on kidney function may be necessary)	Potential for benefit in all patients with sICH, particularly when blood products are contraindicated or declined by patient/family or if cryoprecipitate is not available	Thrombotic complications

*FFP, fresh-frozen plasma; INR, international normalized ratio; PCC, prothrombin complex concentrate; rFVIIa, recombinant factor VIIa; and sICH, symptomatic intracranial hemorrhage*.

## Intracranial Bleeding After Reperfusion Therapy: Risk Factors and Risk Markers

A wide range of risk factors and markers have been demonstrated as being associated with hemorrhagic transformation after reperfusion therapy. We will first describe the risk factors for cerebral hemorrhage demonstrated in RCTs for different reperfusion techniques (IV thrombolysis with alteplase tenecteplase, desmoteplase), MT, intra-arterial thrombolysis, sonothrombolysis. In a first part, we will focus on the hemorrhagic risk reported for each trial, without detailing the efficacy of these different trials. In a second part, we will describe the main risk markers for bleeding that have been described from retrospective studies or large cohorts. Most of the relevant risk factors and markers are summarized in Table 5 with their quantitative association with HT and the population for which this association is applicable.

**Table 5:** Main risk factors and risk markers associated with HT.

	**Study population**	**Bleeding type**	**Odds ratio**
**A. Risk factors from RCT**			
IV thrombolysis (0.9 mg/kg Alteplase) vs. placebo (14, 16)	Any ischemic stroke	sICH	From 9.9^*^ to 10.7^*^
IA prourokinase or urokinase (35)	Large vessel occlusion	sICH	4.4^*^
IV thrombolysis (0.9 mg/kg) vs. placebo (36)	Any ischemic stroke with unknown onset and FLAIR/DWI mismatch on MRI	sICH	5^*^
Mechanical thrombectomy 6-24 h (35, 37–47)	Large vessel occlusion and radiological mismatch	sICH	From 1.75 to 2
Sonothrombolysis + IVT vs. IVT (48)	Any ischemic stroke	sICH	1.47
Use of stent retriever vs. direct aspiration (49)	Large vessel occlusion referred for thrombectomy	sICH	1.23
IV Tenecteplase (0.4 mg/kg) vs. Alteplase (0.9 mg/kg) (50)	Any ischemic stroke	sICH	1.16
IV Tenecteplase (0.25 mg/kg) vs. Alteplase (0.9 mg/kg) (40)	Large vessel occlusion referred for thrombectomy	sICH	1
IV Desmoteplase (62.5–125 μg/kg) vs. Alteplase (0.9 mg/kg) (41)	Any ischemic stroke	sICH	1.5
Mechanical thrombectomy within 6 h (19)	Large vessel occlusion	sICH	1.06
MT + Neretinide vs. MT (51)	Large vessel occlusion	sICH	0.8
MT alone vs. IVT+MT (52)	Large vessel occlusion	sICH	0.7
IVT (0.6 mg/kg) vs. IVT (0.9 mg/kg) (53)	Any ischemic stroke	sICH	0.5^*^
**B. Risk markers from retrospective studies: clinical and biological markers and pre-stroke medication**			
**Baseline**			
Renal Impairment (54, 55)	Any ischemic stroke treated by IVT	Heterogeneous definitions of intracerebral hemorrhage and neurological deterioration	2.79^*^; 2.08^*^
Any antiplatelet agent (54)			2.08^*^
Congestive heart failure (54)			1.96^*^
Atrial fibrillation (54)			1.86^*^
Age (54)			1.78^*^
Statin (54)			1.72^*^
NIHSS (54)			1.55^*^
Diabetes (54)			1.54^*^
Ischemic heart disease (54)			1.54^*^
Prior hypertension (54)			1.5^*^
Glucose (54)			1.10^*^
Smoking (54)			0.7^*^
Triiodothyronine (56)	Any ischemic stroke	ICH	3.46^*^
MMP-9 (57)	Ischemic stroke treated by MT	ICH	2.48^*^
Neutrophil-to-lymphocyte ratio (58)	Any ischemic stroke treated by IVT	sICH	2.08^*^
Fibrinogen decrease (59)	Any ischemic stroke treated by IVT	sICH	1.92^*^
Bilirubin (60)	Ischemic stroke treated by MT	sICH	1.36^*^
NIHSS (61)	Ischemic stroke treated by MT	sICH	1.089^*^ per point
Age (61)			1.028^*^ per year
Onset-to-end procedure time (61)			1.00^*^ per min
Platelet count (54, 62)	Any ischemic stroke treated by IVT	sICH, ICH	From 0.47^*^ to 0.86
Uric acid (63, 64)	Ischemic stroke treated by MT; any ischemic stroke	ICH	From 0.33^*^ to 0.43^*^
Absolute Eosinophil Count ≥ 0.11 × 10^9^/l (65)	Any ischemic stroke treated by IVT	ICH	0.22^*^
**Subacute phase**			
Clinical-genetic (66)	Any ischemic stroke treated by IVT	Parenchymal hemorrhage	5.16^*^
24 h arterial stiffness index (67)	Any ischemic stroke treated by OVT or MT	ICH	1.9^*^
24 h blood pressure variability (68)	Large vessel occlusion referred for MT	sICH	1.71^*^
**C. Risk markers from retrospective studies: imaging**			
Blood-brain barrier permeability (69)	Ischemic stroke treated by IVT or MT	ICH	45.4^*^
Blood-brain barrier disruption after MT (70)	Ischemic stroke treated by MT	ICH	25.3^*^
>10 microbleeds on MRI (71)	Any ischemic stroke treated by IVT	sICH	5.55^*^
MT with ASPECTS 0–4 (72)	Ischemic stroke treated by MT in HERMES	sICH	3.94
Lower ASPECTS score (54)	Any ischemic stroke treated by IVT	Heterogeneous definitions of intracerebral hemorrhage and neurological deterioration	3.46^*^
Visible lesion on CT (54)			2.39^*^
Leukoaraiosis (54)			2.45^*^
ASPECTS score (73)	Ischemic stroke treated by MT	PH	1.87^*^
Intracranial calcifications on CT (74)	Non-cardiogenic ischemic strokes treated by IVT	ICH	1.504^*^
Procedure time (MT) (75)	Ischemic stroke treated by MT	sICH	1.43^*^ per 30 mn
**D. Risk markers from retrospective studies: composite scores**			
	Study population	Bleeding type	Area under the curve
IER-SICH nomogram (61)	Ischemic stroke treated by MT	sICH	0.733^*^
TAG score (76)			0.79^*^
HAT score (77)	Any ischemic stroke treated by IVT without MT	sICH	0.769^*^

*ASPECTS: Alberta Stroke Program Early CT score; CT: Computed Tomography; HAT: Hemorrhage After Stroke; ICH: Intracerebral Hemorrhage; IER-SICH: Italian Registry of Endovascular Stroke Treatment in Acute Stroke Symptomatic Intracerebral Hemorrhage score; IV: Intravenous; IVT: Intravenous Thrombolysis; MRI: Magnetic Resonance Imaging; MT: Mechanical Thrombectomy; PH: Parenchymal Hemorrhage; sICH: symptomatic intracerebral hemorrhage; TAG: TICI-ASPECTS-glucose*.

*Risk markers and risk factors are ordered by odds ratio*.

* *Statistically significant*.

### Risk Factors From IV and IA Thrombolysis RCTs

The NINDS and ECASS III trials were the first to demonstrate the clinical efficacy of IVT. Since the beginning of recanalization trials in acute ischemic stroke, it has been crucial to establish a clear and reproducible definition of cerebral hemorrhage. The most commonly used measure since the ECASS trial has been sICH, defined mostly as a PH2 hematoma with clinical deterioration (usually four points on the NIHSS scale). In the first thrombolysis trials, it seems that alongside the clinical efficacy, patients receiving thrombolysis treatment had a slightly higher risk of developing sICH compared to patients receiving placebo. Biological explanation for this risk seems complex. As previously mentioned, various mechanisms have been described as being involved in reperfusion injuries (4). Moreover, a possible specific explanation for intracerebral hemorrhage after IV alteplase is its role in the upregulation of MMP-9, which has been shown to play an important role in the destruction of the basal lamina (1). In the following section we review HT risks associated with each of the reperfusion therapies used in these trials (Table 5A).

#### Alteplase

Patients included in NINDS (14) received 0.9 mg/kg alteplase (10% as a bolus, max 90 mg), within 3 h of symptom onset. sICH was higher in the tPA group, 6.4 vs. 0.6% (*p* = 0.001). Patients included in ECASS III (13) received 0.9 mg/kg alteplase (10% as a bolus, max 90 mg) within 4.5 h of symptom onset. Intracranial hemorrhage was higher in the alteplase group (27.0 vs. 17.6%, *p* = 0.001). sICH was also higher in the intervention group but was nevertheless low: 2.4 vs. 0.3%, odds ratio, 9.85; 95% CI, 1.26–77.32; *p* = 0.008).

The WAKE UP (36) trial included patients who had an unknown time of symptom onset but a radiological MRI mismatch. Patients received the same dose of tPA as in previous alteplase RCTs. The rate of sICH was 2.0% in the alteplase group vs. 0.4% in the placebo group (*p* = 0.15). This “tissue-based approach,” opposed to “time-based approach,” is consistent with previous retrospective studies that identified early CT signs as strong risk factors of HT in the first IVT trials (78).

The ENCHANTED trial aimed to assess whether low-dose alteplase could reach the same clinical efficacy as a standard dose of alteplase with a decreased hemorrhagic risk (53). Patients were randomized within 4.5 h of symptom onset to receive either a standard 0.9 mg/kg dose or 0.6 mg/kg. In the low-dose group, 1.0% had sICH vs. 2.1% in the standard group (*p* = 0.01). Sixty-three percent of the patients enrolled were Asian [previous studies have shown that Asian people are more likely to present cerebral hemorrhage (79)].

A recent meta-analysis combined five RCTs that compared the efficacy of sonothrombolysis as an adjuvant therapy to IVT (48). The incidence of sICH was 3.8% for the sonothrombolysis group vs. 2.6% for the IVT-alone group with no statistically significant difference.

#### Other IV Thrombolytics

In the NOR-TEST trial (50), patients were randomized to receive alteplase 0.9 mg/kg or tenecteplase 0.25 mg/kg in the 4.5-h window. sICH occurred in 3% of patients in the tenecteplase group vs. 2% of patients in the alteplase group, with no significant difference. These results were confirmed in a recent meta-analysis of five RCTs using tenecteplase vs. alteplase (2.9 vs. 2.6%, not significant) (80). A recent sub-analysis of the NOR-TEST trial confirmed similar risks of hemorrhage among three subgroups: elderly population (37), patients with severe to moderate strokes (38), and patients treated in the 3–4.5 h treatment window (39). Among the RCTs using tenecteplase, the recent EXTEND-IA TNK study (40) is of particular interest because it showed a higher rate of recanalization with tenecteplase in patients with large vessel occlusion addressed for thrombectomy. In this trial, the incidence of sICH was 1% in each group. A recent meta-analysis was conducted to assess the efficacy of intravenous desmoteplase vs. placebo in treating acute ischemic stroke (41). They included six RCTs, including patients 3–9 h after symptom onset. Studies used 62.5 to 125 μg/kg of desmoteplase. sICH was observed in 3.2% of patients in the desmoteplase groups and in 2.1% of placebo-treated patients (no significant difference). Three trials aimed to demonstrate the efficacy of intra-arterial therapy in the 6-h window for patients with middle cerebral artery occlusion. They used recombinant pro-urokinase 6 mg in PROACT I (18), 9 mg in PROACT II (42), and urokinase (maximal dose of 600,000 UI) in the MELT trial (43). A meta-analysis of the three trials (35) demonstrated a higher incidence of sICH in the intervention group: 10.5 vs. 2.4% (*p* = 0.02).

### Risk Factors From Mechanical Thrombectomy RCTs

MT has recently shown great clinical efficacy in acute ischemic stroke with large vessel occlusion (LVO). When describing intracerebral hemorrhage, it is important to understand that the population of patients with LVO may differ from the patient populations included in the IVT trials seen above. IVT trials mainly select patients based on a non-contrast CT scan in order to exclude hemorrhage. This selection means that IVT trials may recruit patients with LVO, but not every patient in an IVT trial has LVO, unlike in MT trials. Patients with LVO present with poorer prognosis and high risk of spontaneous hemorrhage. No MT RCTs have demonstrated a significant increased risk of sICH.

In 2015, the first five RCTs to study MT demonstrated its clinical efficacy (44, 45, 81–83). These first trials were pooled into a meta-analysis that included patients presenting with LVO with various “symptom-onset to randomization” windows (19). They received either MT plus standard of care or standard of care alone (which included IVT in the majority of cases). The rate of sICH was 4.4% in the intervention group vs. 4.3% in the control group (not significant).

Two studies have been conducted to assess the efficacy of MT beyond the 6-h window in patients selected on a radiological mismatch measure. The DAWN trial (46) included patients who presented in the 6–24 h window with a radiological mismatch defined as a maximum diffusion volume on MRI or ischemia parameter on CT perfusion, which differs according to age. sICH occurred in 6% of the MT group vs. 3% in the control group (no significant difference). DEFUSE 3 enrolled patients in the 6–16 h window with a radiological mismatch assessed by perfusion imaging (CT or MRI). The rate of sICH was 7% in the MT group vs. 4% in the control group (not significant) (47).

A recent MT trial aimed to assess the efficacy of a neuroprotective agent (intravenous Neretinide) as an adjuvant therapy to MT in LVO (51). The trial was negative but showed a clinical effect on the subgroup of patients who did not receive alteplase. The rate of sICH was not statistically significantly different, with a rate of 3.5% in the intervention group vs. 4.3% in the control group.

The DIRECT MT trial (52) aimed to demonstrate the non-inferiority of MT alone vs. MT plus IVT in LVO. The incidence of sICH was 4.3% in the MT-alone group vs. 6.1% in the MT plus IVT group, not significant.

### Risk Markers for Bleeding From Retrospective Studies

Apart from revascularization RCTs, a wide range of risk markers have been described as being associated with sICH. These markers were mostly described for retrospective cohorts. They can help to guide further research protocols, but they should not lead to a change in practices based on recommendations following clinical data from RCTs. As retrospective studies reflect current practices, these risk markers were mostly described for IVT or MT procedures, in line with current guidelines (84). Because of the numerous risk marker studies in literature, we chose to report only the main ones in Table 5B. We also discuss a selection of the studies in the following section.

#### Epidemiological Markers

Older age has been associated with increased rates of sICH after IVT (54). Interestingly, reperfusion after MT in non-agenarians does not seem to lead to higher sICH rates than control groups of LVO (85).

Blood pressure following recanalization is a complex variable. In non-recanalized patients, high blood pressure could maintain the efficacy of arterial collaterals. However, higher systolic BP is associated with intracerebral hemorrhage and poor outcome after MT (86). Apart from the “raw” BP measure, it seems that BP variability is another important factor. Time rate of systolic blood pressure variation is independently associated with sICH (68). Furthermore, arterial stiffness has been demonstrated to be associated with sICH after IVT (67).

It has been proposed that patients of Asian ethnicity have an increased risk of intracerebral hemorrhage generally, which would put them even more at risk after recanalization. The RADIANT study looked at 916 patients from different ethnicities who underwent different types of recanalization. Chinese ethnicity was not associated with increased intracerebral hemorrhage, except for patients treated with IVT tPA only, with a prediction model from the logistic regression analysis, in association with age, international normalized value, and partial thromboplastin time (87). One meta-analysis evaluated all usual predictive markers of hemorrhagic transformation after IVT, specifically in the Chinese population (88). These markers were age, male sex, diabetes, atrial fibrillation (AF), previous stroke, onset-to-treatment time, NIHSS, infarct size, and ischemic signs of CT. A recent analysis of 1,324 genotypes of patients undergoing IVT led to the development of a clinical-genetic score using two genetic polymorphisms, which was validated on a MT cohort (66) in a Spanish population. These kinds of studies bring hope for more personalized decision making in the future.

Even if diabetes has been associated with poorer outcome after acute ischemic stroke, it has been debated whether these patients experience more sICH after IVT. Diabetes seems to increase hemorrhagic complications in an experimental stroke model (89). However, current clinical data does not support this idea (90), although one retrospective study including patients treated by MT described an association between sICH and diabetes (91).

Renal impairment has been strongly associated with HT after IVT (54), particularly in severe renal impairment (glomerular filtration rate <30 mL/mn) (92). A recent meta-analysis on a very large population of patients confirmed that chronic kidney disease is associated with sICH with either the NINDS or ECASS definition. The association remains for the <30 mL/mn and the <60 mL/mn subgroups (55). Although alteplase is metabolized by the liver, patients with chronic kidney disease present a higher risk of any hemorrhage, caused by endothelial and platelet dysfunction.

Smoking is a debated marker in HT. The apparent reduction of HT after IVT among smokers could be due to a younger age of the population and less advanced atherosclerosis at the time of presentation (54).

Stroke etiology could play an important role in HT. AF has been described as being a risk factor for hemorrhagic transformation (88, 93, 94), possibly because of larger thrombi (3). Infective endocarditis is a cause of spontaneous hemorrhagic transformation (95) and a possible cause of hemorrhage after recanalization. A recent meta-analysis showed better outcomes in these patients when treated by MT compared to IVT (96), with a risk of hemorrhage 4.14 times higher in the IVT group (96). In addition, retrospective data showed that patients with endocarditis who were treated by MT did not experience more sICH than patients with AF treated by MT (8.0 vs. 5.2%). Interestingly, a retrospective study showed different risk factors associated with sICH between large artery atherosclerosis and cardioembolic subtypes of stroke. Lower LDL-C and higher blood glucose were independent risk factors of large artery atherosclerosis, while lower albumin and platelet counts were independent risk factors of cardioembolic stroke (97).

#### Blood Test Markers

Early decrease of fibrinogen after IVT is associated with hemorrhagic sICH (59). Baseline blood glucose level has been described as an independent predictor of hemorrhagic transformation after IVT (54, 57). Postoperative hyperglycemia is associated with sICH after MT (98). A high neutrophil-to-lymphocyte ratio has been described as a predictor of hemorrhagic transformation after IVT (58). It has been demonstrated that an absolute eosinophil count ≥0.11 × 10^9^/L was independently associated with a 78% reduction in the odds of developing hemorrhagic transformation (65). A low platelet count was associated with hemorrhagic transformation in a recent study (62). A previous larger cohort had a similar result; however, it did not reach statistical significance (54). A lower uric acid level is associated with sICH (63, 64). A recent study found that elevated bilirubin is an independent risk factor of sICH after MT (60). A case-control study found that low triiodothyronine syndrome was independently associated with the risk of hemorrhagic transformation, sICH, and severe parenchymal hematoma in patients with ischemic stroke (56). As described above, blood-brain barrier (BBB) permeability is a key factor of HT. A few biological markers that could affect BBB have been described. This is the case for metalloproteinase 9 (MMP-9) (57).

#### Mild Stroke Risk Markers

In mild stroke, defined by initial clinical stroke severity of NIHSS 0–5, two studies found that IVT was not associated with higher rates of sICH (99, 100). Two other studies observed no significant association between MT and sICH in cohorts of patients with mild stroke (101, 102).

#### Baseline Treatments

Ischemic stroke patients receiving anticoagulation treatment have been described as being at risk of hemorrhagic transformation (103). Direct oral anticoagulants have shown to be less associated with sICH than Vitamin K antagonists (104).

#### Neuro Imaging Markers

##### Computed Tomography

As described above, infarct size plays a key role in HT. Infarct core volume measurement can be challenging, which led to the development of a simple quantitative CT score called the Alberta Stroke Program Early CT Score (ASPECTS) (105). The score ranges from 0 to 10 and divides each hemisphere into 10 regions. Each region with early CT sign of ischemia loses 1 point on the scale. The scale has largely been used in RCTs and in retrospective series, even though its intra-rater and inter-rater reliability could not be sufficient (106). It is not surprising that ischemic core graded by ASPECTS is associated with sICH after MT (73). Furthermore, white matter lesions (leukoaraiosis) visible on CT, which can be a marker of small vessel disease, are associated with sICH (107). Calcification volume on CT is a predictor of sICH (74).

CT perfusion at baseline has been used to predict sICH. The authors used various parameters and thresholds, mostly with the aim of assessing blood-brain barrier disruption (108–111). Blood-brain barrier rupture is radiologically assessed by contrast enhancement of ischemic lesions. This radiological finding is associated with sICH (69, 70, 112). Also, post-procedural contrast accumulation after MT is associated with sICH (70, 113). Assessment of cerebral hemorrhage can be difficult after MT because the intra-arterial contrast can be seen in brain parenchyma for 24 h following the procedure. With regard to this problem, hemorrhage can be more accurately diagnosed using MRI (114) or dual energy CT (115, 116).

##### MRI

###### Conventional MRI.

Conventional MRI can reveal several radiological signs associated with sICH. An initial study found that hemorrhagic transformation is associated with high permeability, hypoperfusion, low apparent diffusion coefficient (ADC), and FLAIR hyperintensity (117). The pooled sensitivity was 82% (95% confidence interval 61–93%) and the pooled specificity was 79% (95% confidence interval 71–85%). On the other hand, a second study found that vascular hyperintensities, old infarcts, and diffusion volume abnormalities were associated with sICH, but the only variable with an acceptable discrimination was volume of DWI abnormality. Other studies seem to show that the most predictive parameter is diffusion lesion volume, assessed by volume or DWI-ASPECTS (72, 118, 119).

Remote hemorrhage after ischemic stroke is a difficult question. In a recent MRI study, the authors demonstrated that there was a pre-existing lesion in half of patients presenting with a remote hemorrhage (120).

###### Perfusion MR.

A recent study used arterial spin labeling to analyze perfusion MRI at 24 h post treatment. This technique can assess the relative cerebral blood flow of the ischemic tissue. Using the 25th percentile of this parameter, the authors obtained an independent predictor of PH and PH2 (121).

Microbleeds visualized on the baseline MRI are associated with an increased risk of sICH (122). A retrospective study demonstrated an association with a significant risk of sICH using a cut-off of 10 microbleeds (71).

##### Digital Subtracted Angiography (DSA)

In a retrospective study of MT, the visualization of angiographic blush was associated with sICH (123). In another study, early visualization of the internal cerebral vein on the lateral projection of the DSA was associated with sICH (124).

#### Predictive Scores From Multiple Risk Markers

It is clear that sICH is associated with various causes, and several authors have tried to establish composite scores including the main markers to obtain more precise predictions of hemorrhage: Cappellari (61), TAG Score (76), and Nisar (77).

#### Technical Considerations Associated With Mechanical Thrombectomy

MT is performed with different practices regarding peri-operative antithrombotics depending on the treatment center protocol. Some centers use peri-procedural heparin, a translation from other endovascular procedures, in order to avoid ischemic complications secondary to catheterization. This practice does not seem to be associated with higher rates of sICH according to a retrospective study (125).

In the case of tandem occlusion, defined as an extracranial occlusion in addition to the intracranial occlusion, the surgeon can be forced to treat the extracranial lesion by emergent stenting. Internal carotid stenting is usually performed under double antiplatelet therapy, which is continued for a few months, depending on the center, in order to prevent intra-stent stenosis. Intra-stent stenosis can lead to distal ischemic emboli or proximal occlusions. In the case of emergent carotid artery stenting during a MT procedure, most surgeons start a single antiplatelet therapy by aspirin and add a second antiplatelet agent after the CT at 24 h, in the absence of hemorrhage. This situation has led to the question of an increased risk of hemorrhage in these procedures.

A first study showed that the sICH rate in patients undergoing MT for tandem occlusion was similar to the sICH rate in the MT RCTs (126). Extracranial stenting and use of antiplatelet therapy was not associated with PH or HI. Another study from the same tandem occlusion cohort showed no increased risk of sICH in the case of antiplatelet therapy use during an MT procedure (127).

An ongoing RCT will demonstrate if emergent carotid stenting in tandem lesions is superior to not treating the extracranial lesion (128). Data regarding safety will assess the hemorrhagic risk of this procedure.

Complete recanalization is associated with less risk of sICH (76) and HI (73), probably due to smaller infarcts, as increased infarct size is associated with more HT.

The incidence of sICH was not higher in the stent retriever group vs. the direct aspiration group in the RCT ASTER I (49). However, stent retriever use was associated with higher hemorrhage in a retrospective series (91).

Multiple studies have investigated the link between the number of attempts performed by the surgeon and the rate of sICH; one study found that an increased number of attempts was associated with an increased risk (129), but two others did not find this association (130, 131). In addition, a longer procedure time seems to be associated with sICH (32, 75).

In the case of MT failure, the surgeon may use an adjuvant therapy that has not been tested for this indication by a RCT. A few of these therapies have been reported in small series: intra-arterial tPA (132), intra-arterial Urokinase (133), and intravenous Tirofiban (134, 135). These series did not show significant association between these adjunctive therapies and sICH.

One study investigated patients treated with repeated MT and showed that they had no increased risk of sICH (136).

## Future Directions

### Blood Pressure Management

A recent RCT investigated treating patients who were given IV thrombolysis by intensive blood pressure reduction. The idea was to decrease the incidence of hemorrhagic transformation. The trial was negative for the primary outcome, which was the reduction of disability at 3 months, assessed by mRS scale (137). On the other hand, the intervention group had a significant reduction in the secondary outcome: “any intracranial hemorrhage.” An ongoing RCT (138) will demonstrate if aggressive reduction of BP (<140/90 mmHg) is effective after MT in reducing risk of intracranial hemorrhage. A recent review described hemodynamic parameters following recanalization and pointed out the fact that BP targets may be dependent on individual parameters such as autoregulatory limits and BP trajectories. In light of this data, chronic high BP in patients with severe carotid stenosis may not require treatment, whereas an unusual increase in BP may represent a risk of reperfusion injury (139).

### Development of Low Hemorrhagic Risk Antithrombotic Therapy and Adjunctive Neuroprotective Therapies

As described above, blood-brain barrier disruption carries a high risk of HT. Several molecular processes have been described and could be potential therapeutic targets. MMP-9 has a key role, and a new phosphodiesterase-III inhibitor called cilostazol has been shown to ameliorate tight junction disruption *in vitro* (140). Cilostazol is a new antiplatelet therapy, and its use in a mouse model showed no increase in cerebral hemorrhage (141). Minocyclin can also target MMP-9 and could be an effective neuroprotective agent (142).

Another approach is to target cytotoxic injuries. Glycyrrhizin could inhibit peroxynitrin production and therefore has a neuroprotective effect on the HT cascade (143, 144).

A new antiplatelet therapy called ACT017 is currently being tested and shows a promising antithrombotic effect with no increased risk of hemorrhage (145).

### Neurosurgery

Several trials are testing minimally invasive surgery for spontaneous intracerebral hematomas (146). If these techniques show efficacy, we can hope for translation to hemorrhagic transformation. Decompressive hemicraniectomy has demonstrated superiority in terms of morbidity and morbidity in middle cerebral artery occlusion complicated by malignant infarct (147), but some studies excluded HT causing mass effect. Despite no trials specifically investigating decompressive surgery for HT, the ongoing SWITCH trial aims to evaluate the safety and efficacy of hemicraniectomy in spontaneous intracerebral hemorrhage.

## Discussion

HT after recanalization therapy can have a wide range of outcomes in terms of severity. The most feared consequence is sICH, which is associated with catastrophic outcomes. It seems crucial in current practice to better characterize hemorrhagic transformation, based on clear pathophysiological mechanisms. It is only with a correct understanding of these mechanisms that stroke physicians will be able to prevent HT, make the most effective recanalization decisions, and try to control HT. Among the current recanalization therapies, IVT can increase the rate of HT, particularly in large infarcts. MT has not been shown to increase HT for the indications used in RCTs. Many clinical, biological, and radiological factors have been described as associated with HT. Developments in neuroimaging and use of composite scores could lead to a more personalized approach for HT prediction. The treatment options for sICH are still disappointing, as it seems very difficult to change its clinical course, which leads almost inevitably to a poor outcome.

## Author Contributions

GC, TM, and LB wrote the manuscript. AB provided radiological resources.

## Conflict of Interest

The authors declare that the research was conducted in the absence of any commercial or financial relationships that could be construed as a potential conflict of interest.
