# Supplementary material for: Intracranial Bleeding After Reperfusion Therapy in Acute Ischemic Stroke
Source: Front Neurol. 2021 Feb 9;11:629920. doi: 10.3389/fneur.2020.629920 (PMC7900408; doi:10.3389/fneur.2020.629920)
Supplement: Supplementary file 1 [file Table_1.docx]

**Supplementary tables**

**Supplementary Table 1**

Acronyms of Stroke Randomized Controlled Trials, sorted by alphabetic order

| DAWN | DWI or CTP Assessment with Clinical Mismatch in the Triage of Wake-Up and Late Presenting Strokes Undergoing Neurointervention with Trevo |
| --- | --- |
| DEFUSE 3 | Endovascular Therapy Following Imaging Evaluation for Ischemic Stroke |
| DIRECT MT | Direct Intraarterial Thrombectomy in Order to Revascularize Acute Ischemic Stroke Patients with Large Vessel Occlusion Efficiently in Chinese Tertiary Hospitals: a Multicenter Randomized Clinical Trial |
| ECASS | European Cooperative Acute Stroke Study |
| ENCHANTED | Intensive blood pressure reduction with intravenous thrombolysis therapy for acute ischaemic stroke |
| ESCAPE | Endovascular Treatment for Small Core and Anterior Circulation Proximal Occlusion with Emphasis on Minimizing CT to Recanalization Times |
| EXTEND-IA | Extending the Time for Thrombolysis in Emergency Neurological Deficits — Intra-Arterial |
| EXTEND-IA TNK | Tenecteplase versus Alteplase before Thrombectomy for Ischemic Stroke |
| HERMES | Highly Effective Reperfusion evaluated in Multiple Endovascular Stroke Trials |
| MAST-E | Multicenter Acute Stroke Trial-Europe |
| MAST-I | Multicenter Acute Stroke Trial-Italy |
| MELT | Middle Cerebral Artery Embolism Local Fibrinolytic Intervention Trial |
| MR CLEAN | Multicenter Randomized Clinical Trial of Endovascular Treatment for Acute Ischemic Stroke in the Netherlands |
| NINDS | National Institute of Neurological Disorders and Stroke |
| NOR-TEST | Tenecteplase versus alteplase for management of acute ischaemic stroke |
| PROACT | Prolyse in Acute Cerebral Thromboembolism |
| RESILIENT | EndoVascular Treatment With Stent-retriever and/or Thromboaspiration vs. Best Medical Therapy in Acute Ischemic Stroke |
| REVASCAT | Randomized Trial of Revascularization with Solitaire FR Device versus Best Medical Therapy in the Treatment of Acute Stroke Due to Anterior Circulation Large Vessel Occlusion Presenting within Eight Hours of Symptom Onset |
| THRACE | THRombectomie des Artères CErebrales |
| SWIFT PRIME | Solitaire with the Intention for Thrombectomy as Primary Endovascular Treatment |
| WAKE UP | Efficacy and Safety of MRI-Based Thrombolysis in Wake-Up Stroke |
